# Prevalence of Oral Mucosal Lesions and Relation to Serum Cotinine Levels—Findings from a Cross-Sectional Study in South Africa

**DOI:** 10.3390/ijerph17031065

**Published:** 2020-02-07

**Authors:** Carla Cruvinel Pontes, Usuf Chikte, Faheema Kimmie-Dhansay, Rajiv T. Erasmus, Andre P. Kengne, Tandi E. Matsha

**Affiliations:** 1 Department of Global Health, Stellenbosch University, Cape Town 7505, South Africa; pontescarla@hotmail.com; 2 Division of Epidemiology and Biostatistics, Stellenbosch University, Cape Town 7505, South Africa; faheemakimmie@gmail.com; 3 Division of Chemical Pathology, Stellenbosch University, Cape Town 7505, South Africa; rte@sun.ac.za; 4 Non-Communicable Diseases Research Unit, South African Medical Research Council, Cape Town 7505, South Africa; andre.kengne@mrc.ac.za; 5 Department of Biomedical Sciences, Cape Peninsula University of Technology, Cape Town 7535, South Africa; MatshaT@cput.ac.za

**Keywords:** oral mucosal lesions, non-communicable diseases, oral health

## Abstract

Oral mucosal lesions (OML) can decrease oral health-related quality of life and some have the potential to become malignant. The aim of the present study was to report the prevalence of OML in relation to age, sex, and serum cotinine levels in a population with mixed ancestry from South Africa. This study is part of the Cape Town Vascular and Metabolic Health (VHM) study, conducted between 2014–2016. Trained dental examiners assessed the oral mucosa for the presence of OML according to WHO criteria. In total, 1976 individuals were included in the study, being 1496 females (75.7%) and 480 males (24.3%) with average age of 49.5 years (SD = 15.3). In total, 262 lesions were detected in 252 participants (overall prevalence of 13%). Males had higher prevalence than females (14% vs. 9%, *p* = 0.008). Participants aged 25–34 had the highest prevalence rates (21%). Participants who had cotinine ≥15 ng/mL had higher prevalence of OML as compared to those with <15 ng/mL (15% vs. 5%, *p* < 0.001). Most common lesions were nicotine stomatitis (33%) and leukoplakia (19%). Age, male sex, and higher cotinine levels were associated with increased prevalence of OML.

## 1. Introduction

Oral health plays a fundamental role in the overall health and well-being. Lack of attention to oral health is a serious concern, given that oral diseases are remarkably prevalent, affecting around 3.5 billion people around the world [1]. Dental caries, periodontal disease, tooth loss, oral mucosal lesions (OML), and oropharyngeal cancers constitute the most challenging oral conditions in terms of public health [2]. 

Oral lesions can decrease oral health-related quality of life due local irritation, discomfort, pain, and the potential to interfere with mastication, speech, swallowing, and oral hygiene [3]. The etiology of OML is not completely understood, however, a number of modifiable risk factors are shared between oral diseases and the leading non-communicable diseases (NCDs): Cardiovascular disease, cancer, respiratory diseases, and diabetes [4,5]. The shared risk factors include social determinants of health, exposure to tobacco, unhealthy diet and alcohol consumption. There is a consistent social gradient between the prevalence and severity of oral diseases and socioeconomic status [1,2]. NCDs, that are associated with social determinants of health and oral diseases, contribute to inequity across and within countries [1].

Cotinine constitutes the main nicotine metabolite in tobacco products and it presents a longer half-life when compared to nicotine (10–30 h versus 30 min, respectively), and is considered a dependable biomarker for tobacco exposure [6]. Studies suggest that serum cotinine levels, ranging from 10 to 20 ng/mL, are compatible with active smoking and, in the present study, 15 ng/mL was chosen as the cut-off value to separate smokers from non-smokers, according to previous studies [7,8].

While, most OML are benign and self-limiting, some can be potentially malignant. Leukoplakia, erythroplakia, and oral sub-mucous fibrosis are the most common pre-cancerous lesions [9]. The importance of early detection of pre-cancerous lesions cannot be underestimated, as it is the most important factor affecting morbidity and survival rates [10].

Epidemiological studies on oral lesions provide crucial information on their prevalence, extent and severity, providing the foundation for oral health policies [11]. There is a scarcity of epidemiological studies on OML in Africa. In South Africa, there are few reports on oral lesions and pre-cancerous lesions. In a study on oral cancer in South Africa, the prevalence of oral squamous cell carcinoma was reported to be higher in males with mixed ethnic background [12].

To the best of our knowledge, there are no studies evaluating serum cotinine levels in relation to oral health status in the country. Cotinine is the main metabolite of nicotine, which is an addictive chemical found in tobacco products [13]. Due to its long half-life, serum cotinine has been used as a biomarker for exposure to tobacco smoking [8]. The aim of the present cross-sectional epidemiological study was to report the prevalence of oral mucosal lesions in relation to age, sex, and serum cotinine levels in a population sample from the Belville South area in South Africa.

## 2. Materials and Methods

### 2.1. The Sample

This cross-sectional study was conducted as part of the Cape Town Vascular and Metabolic Health (VHM) study, conducted from 2014 to 2016 in South Africa, as described previously [14]. The Ethics Committee of the Faculty of Health and Wellness Sciences of the Cape Peninsula University of Technology (N14/01/003a) approved the study, which was conducted according to the Declaration of Helsinki. The target population was adults living in the Bellville South area, a community characterized by mixed ethnic ancestry as well as low-socioeconomic status. A consecutive sampling technique was used to recruit potential participants according to the following inclusion criteria: Individuals of mixed ancestry (defined in accordance to the South African Population Registration Act No. 30 of 1950, repealed in 1991), living in the Bellville area of the Western Cape Province, South Africa, with a minimum age of 18 years who signed the informed consent [15]. The exclusion criteria included individuals requiring prophylactic antibiotics, undergoing renal dialysis, with intellectual disabilities, with cancer, younger than 18 years, pregnant women, and those who were too ill or unable to sign the consent. 

### 2.2. Clinical Examination

Trained and calibrated dental examiners comprehensively assessed the oral mucosa in each participant. The oral examination was undertaken using a portable dental chair, a portable overhead LED light, dental mirrors, and the handle of a periodontal probe for tissue retraction. All sections of the oral cavity were inspected systematically for any variation from normal in the following sequence, according to the criteria from the WHO: (1) Upper and lower labial mucosa and labial sulci, (2) buccal mucosa, (3) tongue, (4) floor of the mouth, (5) palate, and (6) gingiva and alveolar [16] The presence of Malignant tumor (oral cancer), leukoplakia, lichen planus, ulceration, acute necrotizing ulcerative gingivitis (ANUG), candidiasis, abscess and other condition was recorded for each of the following locations: Vermillion border, commissures, lips, sulci, buccal mucosa, floor of the mouth, tongue, soft and hard palate, gingiva, and alveolar ridge.

### 2.3. Serum Cotinine

In the present study, 15 ng/mL was used as the cut-off value to differentiate smokers from non-smokers, based on previous studies [6,7,8]. A fasting blood sample was collected and transported to an accredited laboratory within 6 h of collection for processing. A chemiluminescent assay was used after blood samples had been processed, in order to measure serum cotinine levels (Immulite 1000, Siemens), as described previously [15].

### 2.4. Statistical Analysis

Data were analyzed through the statistical package Stata 15.1 (StataCorp, LLP, College Station, TS, USA). Presence of oral mucosal lesions was presented as percentage prevalence. Age was categorized into groups (≤24, 25–34, 35–44, 45–54, 55–64, and ≥65 years), as well as serum cotinine levels (<15 ng/mL and ≥15 ng/mL). The Chi-square test and the Fisher test were used to compare the proportion of oral lesions in relation to sex, age group, and cotinine categories. A logistic regression was carried out to determine the probability of having at least one lesion of any type given sex, age group, and cotinine levels. A *p*-value of 0.05 and below was considered statistically significant.

## 3. Results

### 3.1. Total Prevalence

In total, 1976 individuals were included in the study, from which 1496 were females (75.7%) and 480 were males (24.3%). The sample had an average age of 49.5 years (SD = 15.3), ranging from 18 to 91 years. The average age was 47.3 for males and 50.2 years for females.

**Figure 1:**
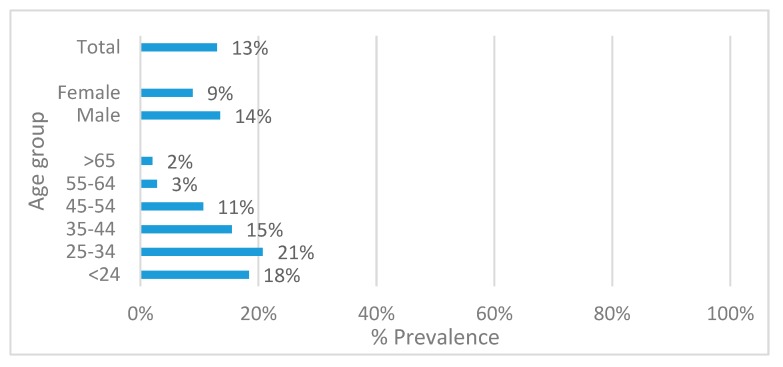
Total prevalence of oral mucosal lesions according to sex (*p* = 0.003), age group (*p* < 0.001), and total.

A total of 262 lesions were detected in 252 participants (overall prevalence of 13%, Figure 1). OML were associated with sex, with males presenting higher prevalence of lesions than females (14% versus 9%, respectively, *p* = 0.003, Figure 1). 

There was an association between age-group and the prevalence of OML (*p* < 0.001, Figure 1). The highest prevalence of OML was observed in the age group 25–34 years (21%), followed by the <24 (18%) and the 35–44 age group (15%). Participants aged 55 years and older had the lowest prevalence of lesions (2–3%).

**Figure 2:**
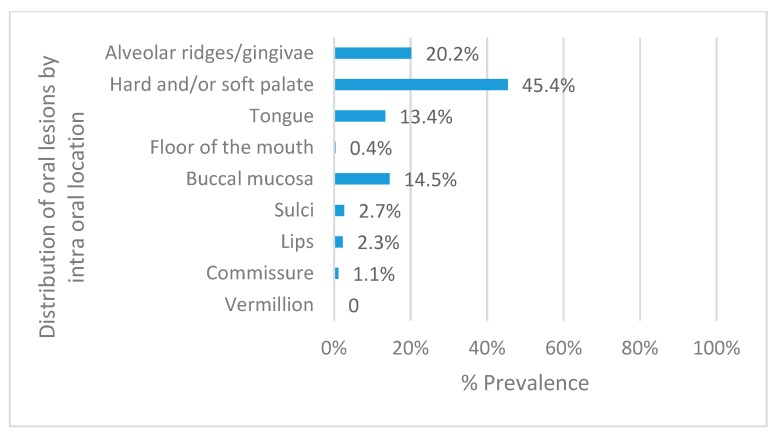
Total prevalence of oral mucosal lesions by intra-oral location.

The three most frequent locations for the OML were the palate (45.4%), the alveolar ridge, and gingiva (20.2%) and the buccal mucosa (14.5%, Figure 2). The most common lesion in the palate was nicotine stomatitis (66.4%); in the alveolar ridge/gingiva it was leukoplakia (35.8%); and in the buccal mucosa was abscess (78.9%). 

An association was observed between serum cotinine categories and total prevalence of oral lesions. Participants who had serum cotinine levels ≥15 ng/mL had higher prevalence of OML as compared to those with levels below 15 ng/mL (15% versus 5%, respectively, *p* < 0.001). Serum cotinine ≥15 ng/mL was observed in 59.7% of all men and in 45.6% of all women (*p* < 0.01).

The association between the prevalence of lesions and sex, age group, and cotinine levels remained significant in the regression analysis (Table 1). Females had lower prevalence of OML in comparison to males (OR = 0.395, 95% CI: 0.297–0.525, *p* < 0.001); the odds of having lesions in males were 2.5 times greater in comparison to females. Participants older than 54 years had lower prevalence of lesions in comparison to the younger age groups. Subjects with cotinine levels greater than 15 ng/mL had a higher chance of having lesions, in comparison to those who had levels below 15 ng/mL (OR = 2.938, 95% CI: 2.165–3.987, *p* < 0.001).

### 3.2. Oral Mucosal Lesions (OML) Lesion Types

Nicotine stomatitis was the most prevalent lesion (33% of all lesions, *n* = 87, Table 2), followed by leukoplakia (19%, *n* = 48), abscess (14%, *n* = 35), candidiasis (6%, *n* = 12), ulcerations (3%, *n* = 9), and ANUG (3%, *n* = 7). In total, 21% of the oral lesions were classified as ‘other conditions’.

### 3.3. Nicotine Stomatitis

The overall prevalence of nicotine stomatitis was 4.4%. Sex was not associated with prevalence of this lesion (*p* = 0.63, Table 2). There was an association between age group and nicotine stomatitis (*p* < 0.001) and the age groups with the highest prevalence were 25–34 years (30%), 35–44 years (25%), and 45–54 years (28%). Most nicotine stomatitis lesions were located in the palate (91%, *n* = 79), followed by alveolar ridge/gingiva (5%, *n* = 4), lips (2%, *n* = 2), sulci (1%, *n* = 1) and tongue (1%, *n* = 1).

**Figure 3:**
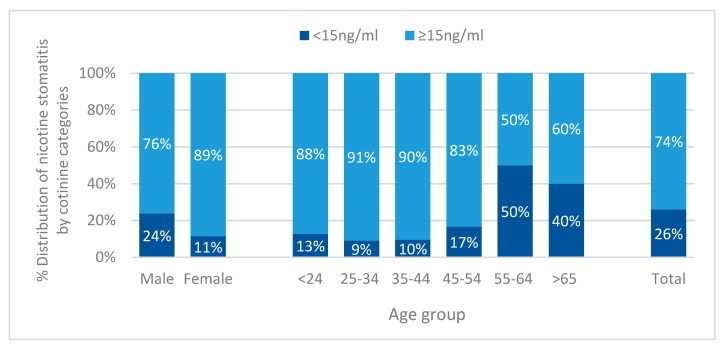
Distribution of nicotine stomatitis according to serum cotinine categories (<15 ng/mL and ≥15 ng/mL) in relation to sex (*p* = 0.03 for males, *p* < 0.001 for females), age group (*p* = 0.009 for 25–34, *p* = 0.002 for 35–44 and *p* = 0.001 for 45–54), and total (*p* < 0.001).

Cotinine categories were associated with prevalence of nicotine stomatitis (*p* < 0.001, Figure 3). Regarding distribution of nicotine stomatitis by cotinine levels, participants with level 15 ng/mL and above had about three-time more lesions than participants with serum cotinine ≥15 ng/mL. This association was statistically significant for males (*p* = 0.03) and females *(p* < 0.001), as well as for the age groups 25–34 (*p* = 0.009), 35–44 (*p* = 0.002), and 45–54 (*p* = 0.001, Figure 3). 

### 3.4. Leukoplakia

**Figure 4:**
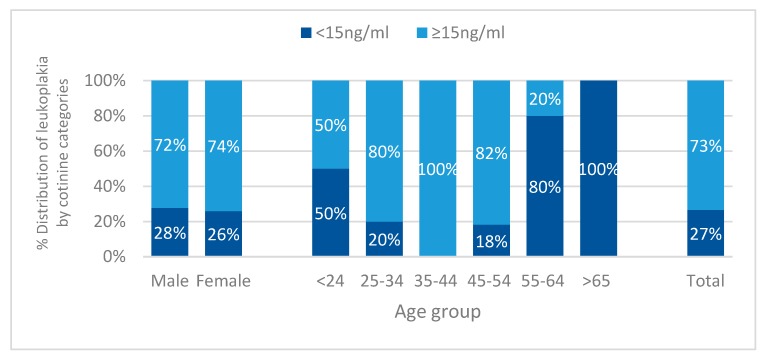
Distribution of leukoplakia according to serum cotinine categories (<15 ng/mL and ≥15 ng/mL) in relation to sex (*p* = 0.007 for females), age group (*p* = 0.01 for 35–44), and total (*p* < 0.001).

Oral leukoplakia was more prevalent in females (56%) as compared to males (44%, *p* = 0.001, Table 2), with total prevalence of 2.4%. There was an association between age group and prevalence of oral leukoplakia (*p* < 0.001), with participants aged 25–34 presenting the highest prevalence (35%, Figure 4).

Tongue (37.3%, *n* = 19) and alveolar ridge (37.3%, *n* = 19) were the most common locations for leukoplakia lesions, followed by palate (9.8%, *n* = 5), sulci (5.9%, *n* = 3), lips (5.9%, *n* = 3), and buccal mucosa (3.9%, *n* = 2). 

Cotinine categories were associated with the distribution of leukoplakia (*p* < 0.001, Figure 4). Female participants with serum cotinine 15 ng/mL and above had higher prevalence of leukoplakia when compared to females with cotinine levels below 15 ng/mL (*p* = 0.007). This association was also statistically significant for the age group 35–44, with all leukoplakia lesions being present in participants with cotinine levels ≥15 ng/mL (*p* = 0.01). 

### 3.5. Candidiasis, Abscess, Ulceration, Acute Necrotizing Ulcerative Gingivitis (ANUG), and other Conditions

The overall prevalence of candidiasis was 6% and it was not associated with sex or age group (Table 2). Candida lesions were observed in the palate (63%, *n =* 10), alveolar ridges/gingiva (25%, *n =* 4), tongue (6%, *n =* 1) and sulci (6%, *n =* 1). The prevalence of candidiasis was higher in subjects who presented serum cotinine levels 15 ng/mL and above, when compared to those with cotinine level below 15 ng/mL (83% versus 17%, respectively, *p* = 0.02). 

Prevalence of abscess (15%) was not associated with sex or cotinine levels. It was associated with age group (*p* < 0.001), with participants aged 45-54 presenting the highest prevalence (29%). In terms of location, 83% (*n =* 30) of the abscesses were located in the alveolar ridge/gingiva, followed by 8% (*n =* 3) in the buccal mucosa, 6% (*n =* 2) in the palate and 3% (*n =* 1) in the commissure.

The prevalence of ulceration (3%) and ANUG (3%) were not associated with sex, age group, or cotinine categories. Ulcerations were mostly observed at the buccal mucosa (56%, *n =* 5), sulci (22%, *n =* 2), floor of the mouth (11%, *n =* 1), and tongue (11%, *n =* 1).

Lesions classified as “other conditions” were not associated with sex or cotinine levels. They were associated with age group (*p* = 0.002), with the highest prevalence in the 35–44 age group (31%). The palate was the most frequent location (41%, *n =* 23), followed by alveolar ridge/gingiva (29%, *n =* 16), tongue (23%, *n =* 13), commissure (4%, *n =* 2), lips (2%, *n =* 1), and sulci (2%, *n =* 1).

## 4. Discussion

To our knowledge, this is the first epidemiological study to report on the prevalence of oral mucosal lesions in South Africa since the last national survey 30 years ago. Results from our study show total prevalence of OML of 13%, with males presenting higher prevalence than females. Ages between 25–34 years and serum cotinine levels that indicate smoking (≥15 ng/mL) were associated with higher prevalence of OML. Nicotine stomatitis was the most prevalent condition, corresponding to one-third of all identified lesions.

To our knowledge, this is the first epidemiological population study to present the prevalence of oral mucosal lesions in this South African sample of adults with mixed ethnic background. Comparison with previous studies is not possible as data are only available for HIV-related oral lesions [17,18,19,20], lesions in children [21], and in pregnant women [22].

A variety of studies from other countries report prevalence of OML, ranging from 2 to 80% [23,24,25,26,27]. Some studies include normal variations of the oral mucosa, such as hairy tongue, fissured tongue, and bone exostosis, hence, there are differences in the definition of oral lesions can partially explain the high rates reported by some authors [28]. The prevalence of oral lesions in South Africa (13%) is comparable to other upper middle income countries, such as Turkey (15.5%) [26], China (9.5%) [24], and Malaysia (9.7%) [28], and somewhat lower than the prevalence of oral lesions in Brazil (23,3%) [29].

The higher prevalence of oral lesions in male participants could be explained by higher serum cotinine levels. In the literature, there are studies reporting no association of OML with sex [29,30], as well as studies reporting higher prevalence of oral lesions in males [5,26] and females [31]. Besides exposure to tobacco, alcohol consumption is another risk factor for OML and other NCDs. Studies have reported high risk for harmful drinking among South Africans with mixed ethnic background from underprivileged communities [32,33]. In future studies on NCDs and oral diseases, information on drinking habits can be useful when planning strategies to address common risk factors to NCDs. 

Age has been associated with an increased prevalence of OML [5,34,35]. However, this was not the case in the studied sample, as most lesions were observed in participants aged 25 to 34 years, with decreased prevalence for participants 55 and older. A high exposure to tobacco, early in adulthood in the current sample, and the potential exposure to secondary smoking within each household included in the study, might predispose to oral lesions earlier in life [36].

Almost one fifth of oral lesions observed in this study were identified as leukoplakia, and the estimated risk for malignant transformation for these lesions range from 0.13 to 34% [37]. Early screening and diagnosis is still the best approach to decreasing morbidity and mortality associated with oral cancer [38], which further highlights the need for oral health programs that promote screening, diagnosis, and treatment of lesions in the oral cavity. 

Smoking is a strong risk factor for NCDs, oral cancer, and OML [39], In the current study, there was an association between the prevalence of OML and increased cotinine levels, particularly for leukoplakia, which is potentially pre-cancerous, and for nicotine stomatitis, which is directly related to exposure to tobacco. Creation of robust tobacco cessation programs needs to become a priority for this disadvantaged population group in terms of prevention and management of oral diseases and other NCDs [40].

### Limitations of the Study

The high prevalence of non-diagnosed lesions can point to a lack of precise diagnostic criteria. Examiners were not capable of differentiating between 21% of the mucosal changes encountered. Future studies should include diagnostic tests and use digital resources to facilitate the interaction with a specialist in oral medicine during clinical examinations for diagnostic accuracy. The clinical examination took place during weekdays, which have resulted in selection bias, due to exclusion of males who worked.

Although, patients with leukoplakia were referred to an appropriate health-care facility for further laboratory investigations to confirm the initial clinical diagnosis, and they were not followed-up. Hence, the initial tentative diagnosis was not confirmed through biopsy nor other diagnostic tests. An epidemiological study from Japan reported mass screening for leukoplakia in 3131 participants. The authors reported a positive predictive value of 0.73 and false positive ratio of 0.27 for diagnosing oral leukoplakia during the primary mass screening, which can be extrapolated to the results from the current study [41].

## 5. Conclusions

This is the first study providing epidemiological data on oral lesions and cotinine levels for a population sample of mixed ethnic background from South Africa. Males, participants younger than 54 years, and higher cotinine levels were associated with a higher prevalence of lesions. The two most common lesions were nicotine stomatitis and leukoplakia, the latter has the potential to undergo malignant transformation. Programs aimed at reducing exposure to tobacco should be an integral part of reducing the burden of NCDs in this population group. Early screening and diagnosis of mucosal lesions should be a priority in primary care settings, as it can decrease morbidity and mortality related to oral cancer. 

## Figures and Tables

**Table 1:** Association of Oral Mucosal Lesions and gender, age category, and cotinine levels.

Variable	Odds Ratio	95% Confidence Interval	*p*-Value
**Gender**			
Male	Reference		
Female	0.395	0.297–0.525	0.000 *
**Age group (years)**			
≤24	Reference		
2–34	1.125	0.65–1.941	0.671
3–44	1.390	0.81–2.378	0.230
4–54	0.607	0.35–1.041	0.070
5–64	0.247	0.13–0.460	0.000 *
≥65	0.171	0.08–0.358	0.000*
**Cotinine Levels**			
<15 ng/mL	Reference		
≥15 ng/mL	2.938	2.16–3.987	0.000 *

* *p*-Value < 0.05 (Statistically significant at 5% level of significance).

**Table 2:** Distribution (% and count) of different oral mucosal lesions according to sex and age group.

	Nicotine Stomatitis	Leukoplakia	Abscess	Candidiasis	Ulceration	ANUG	Other
**Sex**							
Male	26% (23)	44% (21)	34% (12)	25% (3)	44% (4)	29% (2)	24% (13)
Female	74% (64)	56% (27)	66% (23)	75% (9)	56% (5)	71% (3)	76% (41)
*p*-Value	0.63	0.001	0.164	1.00	0.233	0.680	0.970
**Age group**							
<24	9% (8)	8% (4)	20% (7)	8% (1)	22% (2)	14% (1)	2% (1)
25–34	30% (26)	35% (17)	26% (9)	24% (3)	34% (3)	30% (2)	22% (12)
35–44	25% (22)	19% (9)	17% (6)	33% (4)	22% (2)	14% (1)	31% (17)
45–54	28% (24)	25% (12)	28% (10)	25% (3)	11% (1)	14% (1)	20% (11)
55–64	2% (2)	11% (5)	9% (3)	8% (1)	11% (1)	14% (1)	16% (8)
>65	6% (5)	2% (1)	0	0	0	14% (1)	9% (5)
*p*-Value	<0.001	<0.001	<0.001	0.129	0.068	0.704	0.002
**Total**	33% (87)	19% (48)	14% (35)	7% (12)	3% (9)	3% (7)	21% (54)

*ANUG:* acute necrotizing ulcerative gingivitis.

## References

[1] Peres M.A., Macpherson L.M.D., Weyant R.J., Daly B., Venturelli R., Mathur M.R., Listl S., Keller Celeste R., Guarnizo-Herreño C.C., Kearns C. (2019). Oral Health 1 Oral diseases: A global public health challenge. Lancet.

[2] Petersen P.E., Bourgeois D., Ogawa H., Estupinan-Day S., Ndiaye C. (2005). The global burden of oral diseases and risks to oral health. Bull. World Health Organ..

[3] Yamane-Takeuchi M., Ekuni D., Mizutani S., Kataoka K., Taniguchi-Tabata A., Azuma T., Furuta M., Tomofuji T., Iwasaki Y., Morita M. (2016). Associations among oral health-related quality of life, subjective symptoms, clinical status, and self-rated oral health in Japanese university students: A cross-sectional study. BMC Oral Health.

[4] Shulman J.D., Beach M.M., Rivera-Hidalgo F. (2004). The prevalence of oral mucosal lesions in U.S. adults. J. Am. Dent. Assoc..

[5] Garcia-Pola Vallejo M.J., Martinez Diaz-Canel A.I., Garcia Martin J.M., Gonzalez Garcia M. (2002). Risk factors for oral soft tissue lesions in an adult Spanish population. Community Dent. Oral Epidemiol..

[6] Benowitz N.L., III P.J., Ahijevych K., Jarvis M.J., Hall S., LeHouezec J., Hansson A., Lichtenstein E., Henningfield J., Tsoh J. (2002). Biochemical verification of tobacco use and cessation. Nicotine Tob. Res..

[7] Jarvis M.J., Tunstall-Pedoe H., Feyerabend C., Vesey C., Saloojee Y. (1987). Comparison of tests used to distinguish smokers from nonsmokers. Am. J. Public Health.

[8] Bunaes D.F., Mustafa M., Mohamed H.G., Lie S.A., Leknes K.N. (2017). The effect of smoking on inflammatory and bone remodeling markers in gingival crevicular fluid and subgingival microbiota following periodontal therapy. J. Periodontal Res..

[9] Van der Waal I. (2009). Potentially malignant disorders of the oral and oropharyngeal mucosa; terminology, classification and present concepts of management. Oral Oncol..

[10] Sreekumar V.N. (2019). Global Scenario of Research in Oral Cancer. J. Maxillofac. Oral Surg..

[11] Gheno J.N., Martins M.A.T., Munerato M.C., Hugo F.N., Sant’ana Filho M., Weissheimer C., Carrard V.C., Martins M.D. (2015). Oral mucosal lesions and their association with sociodemographic, behavioral, and health status factors. Braz. Oral Res..

[12] Botha P.J., Schoonees A., Pontes C.C. (2018). Mapping oral cancer research in South Africa. S. Afr. Dent. J..

[13] Al-bayaty F., Baharuddin N., Abdulla M. (2010). The relationship between serum cotinine levels and periodontal status. OnLine J. Biol. Sci..

[14] Matsha T.E., Hassan M.S., Kidd M., Erasmus R.T. (2018). The 30-year cardiovascular risk profile of South Africans with diagnosed diabetes, undiagnosed diabetes, pre-diabetes or normoglycaemia: The Bellville, South Africa pilot study. Cardiovasc. J. Afr..

[15] Matsha T.E., Soita D.J., Hassan M.S., Hon G.M., Yako Y.Y., Kengne A.P., Erasmus R.T. (2013). Three-year’s changes in glucose tolerance status in the Bellville South cohort: Rates and phenotypes associated with progression. Diabetes Res. Clin. Pract..

[16] WHO (2016). Oral Health Surveys: Basic Methods.

[17] Arendorf T.M., Bredekamp B., Cloete C.A.C., Sauer G. (2007). Oral manifestations of HIV infection in 600 South African patients. J. Oral Pathol. Med..

[18] Bajomo A.S., Ayo-Yusuf O.A., Rudolph M.J., Tsotsi N.M. (2013). Impact of oral lesions among South African adults with HIV/AIDS on oral health-related quality of life. J. Dent. Sci..

[19] Naidoo S., Chikte U., Gouws E., Abdool-Karim S. (2009). Oral mucosal lesions and HIV status in a rural household survey in South Africa. SADJ.

[20] Lambert R.F., Orrell C., Haberer J.E. (2017). “It was pain. That’s it. It was pain.” Lack of oral health care among otherwise healthy young adults living with HIV in South Africa: A qualitative study. PLoS ONE.

[21] Arendorf T.M., Ross R. (1996). Oral soft tissue lesions in a black pre-school South African population. Community Dent. Oral Epidemiol..

[22] Africa C.W.J., Turton M. (2019). Oral Health Status and Treatment Needs of Pregnant Women Attending Antenatal Clinics in KwaZulu-Natal, South Africa. Int. J. Dent..

[23] Reichart P.A. (2000). Oral mucosal lesions in a representative cross-sectional study of aging Germans. Community Dent. Oral Epidemiol..

[24] Lin H.C., Corbet E.F., Lo E.C.M. (2001). Oral Mucosal Lesions in Adult Chinese. J. Dent. Res..

[25] Espinoza I., Rojas R., Aranda W., Gamonal J. (2003). Prevalence of oral mucosal lesions in elderly people in Santiago, Chile. J. Oral Pathol. Med..

[26] Cebeci A.-R.-I., Gülşahi A., Kamburoglu K., Orhan B.-K., Oztaş B. (2009). Prevalence and distribution of oral mucosal lesions in an adult Turkish population. Med. Oral Patol. Oral Cir. Bucal.

[27] Do L., Spencer A., Dost F., Farah C. (2014). Oral mucosal lesions: findings from the Australian National Survey of Adult Oral Health. Aust. Dent. J..

[28] Zain R.B., Ikeda N., Razak I.A., Axell T., Majid Z.A., Gupta P.C., Yaacob M. (1997). A national epidemiological survey of oral mucosal lesions in Malaysia. Community Dent. Oral Epidemiol..

[29] Correa M.B., Tarquinio S.B.C., de Oliveira L.J.C., Peres M.A., Peres K.G., Gigante D.P., Horta B.L., Demarco F.F. (2013). Factors associated with prevalence of oral lesions and oral self-examination in young adults from a birth cohort in Southern Brazil. Cad. Saude Publica.

[30] El Toum S., Cassia A., Bouchi N., Kassab I. (2018). Prevalence and Distribution of Oral Mucosal Lesions by Sex and Age Categories: A Retrospective Study of Patients Attending Lebanese School of Dentistry. Int. J. Dent..

[31] Kansky A.A., Didanovic V., Dovsak T., Brzak B.L., Pelivan I., Terlevic D., Pelivan I., Terlevic D. (2018). Epidemiology of oral mucosal lesions in Slovenia. Radiol. Oncol..

[32] Al-Mobeeriek A., AlDosari A.M. (2009). Prevalence of oral lesions among Saudi dental patients. Ann. Saudi Med..

[33] Peltzer K., Davids A., Njuho P. (2011). Alcohol use and problem drinking in South Africa: Findings from a national population-based survey. Afr. J. Psychiatry.

[34] Vellios N.G., Van Walbeek C.P. (2017). Self-reported alcohol use and binge drinking in South Africa: Evidence from the National Income Dynamics Study, 2014–2015. S. Afr. Med. J..

[35] Cueto A., Martínez R., Niklander S., Deichler J., Barraza A., Esguep A. (2013). Prevalence of oral mucosal lesions in an elderly population in the city of Valparaiso, Chile. Gerodontology.

[36] Jainkittivong A., Aneksuk V., Langlais R.P. (2002). Oral mucosal conditions in elderly dental patients. Oral Dis..

[37] World Health Organization (2017). WHO Monograph on Tobacco Cessation and Oral Health Integration.

[38] Rhodus N.L., Kerr A.R., Patel K. (2014). Oral Cancer. Dent. Clin. N. Am..

[39] Hille J., Johnson N.W. (2017). The burden of oral cancer in sub-Saharan Africa. Transl. Res. Oral Oncol..

[40] Barnes L., Universitäts Spital Zürich, Departement Pathologie, International Academy of Pathology, World Health Organization, International Agency for Research on Cancer (2005). Pathology and Genetics of Head and Neck Tumours.

[41] Nugent R., Bertram M.Y., Jan S., Niessen L.W., Sassi F., Jamison D.T., Pier E.G., Beaglehole R. (2018). Investing in non-communicable disease prevention and management to advance the Sustainable Development Goals. Lancet.

